# Musculoskeletal Sarcoidosis With Sacroiliac Involvement: Report of a Rare Case

**DOI:** 10.7759/cureus.99759

**Published:** 2025-12-21

**Authors:** Mohamed Nassiri, Abdessamad Laaribi, Abdessalam Achkoun, Rachid Chafik

**Affiliations:** 1 Department of Orthopedics and Traumatology A, Ibn Tofail Hospital, Mohammed VI University Hospital Center (CHU Mohammed VI), Marrakech, MAR; 2 Faculty of Medicine and Pharmacy of Marrakech, Cadi Ayyad University, Marrakech, MAR; 3 Department of Orthopedics and Traumatology A, Ibn Tofail Hospital, Mohammed VI University Hospital Center (CHU Mohammed VI), Marrakesh, MAR

**Keywords:** bone lesion, musculoskeletal sarcoidosis, pseudotumoral myositis, sacroiliitis, sarcoidosis

## Abstract

Sarcoidosis is a systemic granulomatous disease of unknown etiology that rarely affects the musculoskeletal system. Sacroiliac involvement is exceptional and may mimic spondyloarthropathies or malignancy. We report the case of a 47-year-old man presenting with progressive bilateral sacroiliac and shoulder pain. MRI showed bilateral sacroiliitis with diffuse bone marrow lesions in the pelvic girdle and femoral heads, as well as a pseudotumoral lesion of the iliopsoas muscle. CT demonstrated a lytic lesion of the left humeral head with cortical rupture. Laboratory findings revealed elevated C-reactive protein, hypercalciuria, and increased serum angiotensin-converting enzyme (ACE) levels. Histopathological examination of bone and muscle biopsies demonstrated non-caseating granulomas. The diagnosis of musculoskeletal sarcoidosis was established. The patient was treated with oral prednisone (1 mg/kg/day), leading to marked clinical improvement. Sacroiliac sarcoidosis is an unusual presentation requiring careful exclusion of infectious and inflammatory disorders. MRI is useful for detecting bone and muscle lesions, but histological confirmation remains essential. Corticosteroid therapy is effective in most cases, though close monitoring is required.

## Introduction

Sarcoidosis is a multi-system granulomatous disorder of unknown etiology, most commonly affecting the lungs and lymph nodes, but virtually any organ system may be involved. The disease is characterized histologically by non-caseating granulomas and displays a wide spectrum of clinical presentations, ranging from asymptomatic forms to severe, multi-system disease [1].

Musculoskeletal involvement is reported in approximately 1-13% of patients and may manifest as acute or chronic arthritis, periarticular soft tissue inflammation, tenosynovitis, myopathy, or osseous lesions [1]. While acute arthritis is relatively well recognized, bone and muscle involvement remain uncommon and are often underdiagnosed due to their nonspecific clinical and radiological features. Osseous sarcoidosis may present with lytic, sclerotic, or mixed lesions that can closely resemble metastatic disease, infection, or other inflammatory conditions, frequently leading to diagnostic delay.

Sacroiliac joint involvement is particularly rare, with an estimated prevalence of about 6% among patients with sarcoidosis. When present, sacroiliac sarcoidosis may clinically and radiologically mimic ankylosing spondylitis, tuberculosis, or neoplastic disease, making differential diagnosis challenging. Imaging modalities such as MRI are helpful for detecting inflammatory and infiltrative lesions, but findings are not specific, and histological confirmation is often required to establish the diagnosis.

Muscle sarcoidosis is usually asymptomatic and frequently discovered incidentally, although symptomatic forms such as chronic myopathy or pseudo-tumoral myositis have been described. The pseudo-tumoral form is exceptionally rare and may simulate soft tissue tumors on imaging, further complicating diagnosis [2-3].

Herein, we present a rare case of musculoskeletal sarcoidosis with concomitant sacroiliac involvement, diffuse osseous lesions, and pseudo-tumoral muscular involvement, highlighting the diagnostic challenges and the importance of histopathological confirmation.

## Case presentation

A 47-year-old man with no prior medical history presented with a six-month history of progressive bilateral sacroiliac and shoulder pain. Initial symptoms included fever, chills, and weight loss, which resolved spontaneously within two weeks, leaving persistent articular pain. The patient subsequently developed transient cervical and inguinal lymphadenopathy.

**Figure 1:**
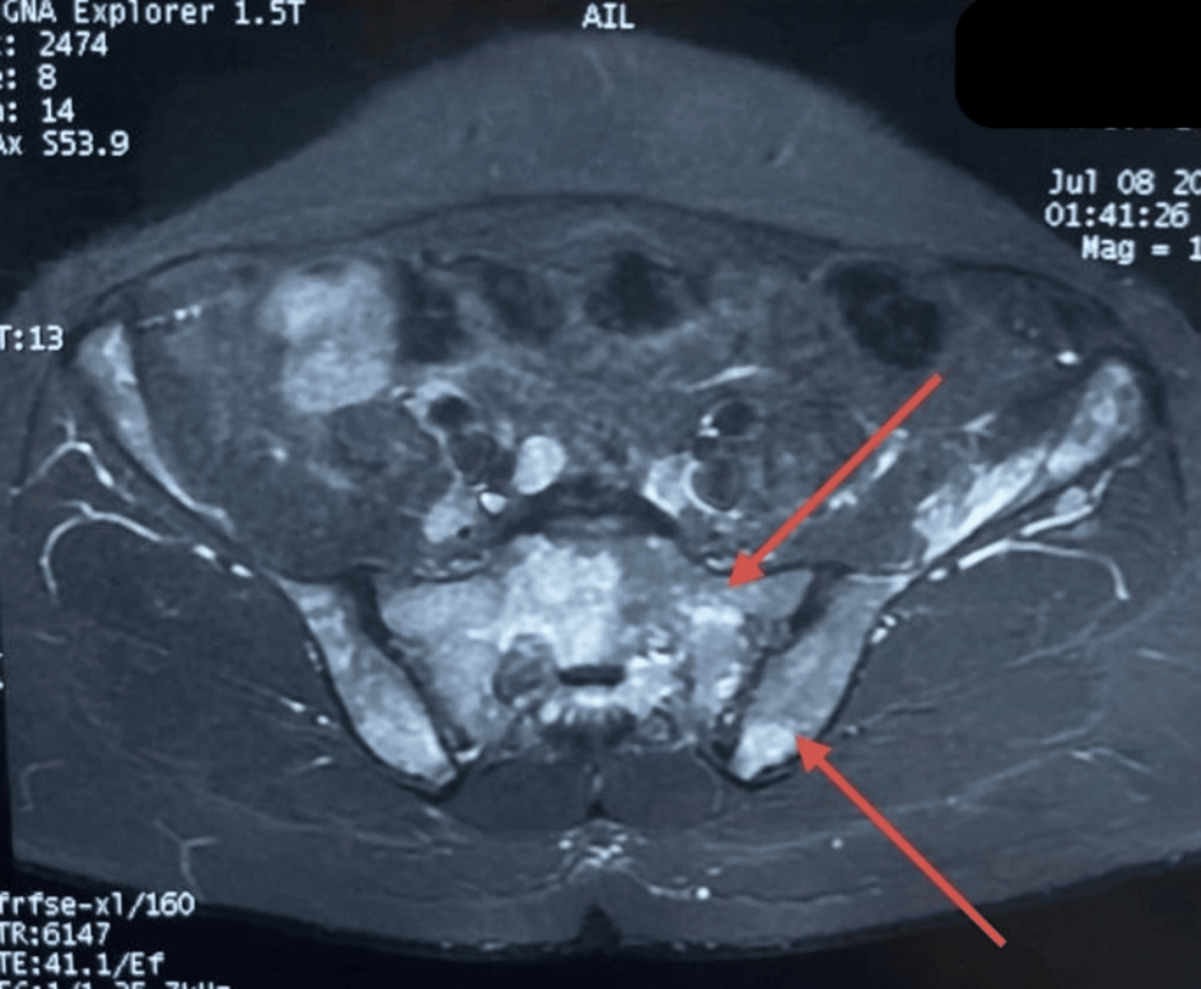
Pelvic MRI Pelvic MRI (coronal STIR sequence) showing bilateral sacroiliac arthritis with diffuse bone marrow hyperintensity involving the pelvic girdle and femoral heads. Cortical integrity is preserved. STIR - short tau inversion recovery

Pelvic MRI revealed bilateral sacroiliac arthritis with diffuse bone marrow signal abnormalities involving the pelvic girdle and femoral heads, hyperintense on short tau inversion recovery (STIR) sequences with preserved cortical integrity. A poorly defined lobulated lesion of the left iliopsoas muscle (53 mm) was also detected (Figure 1).

**Figure 2:**
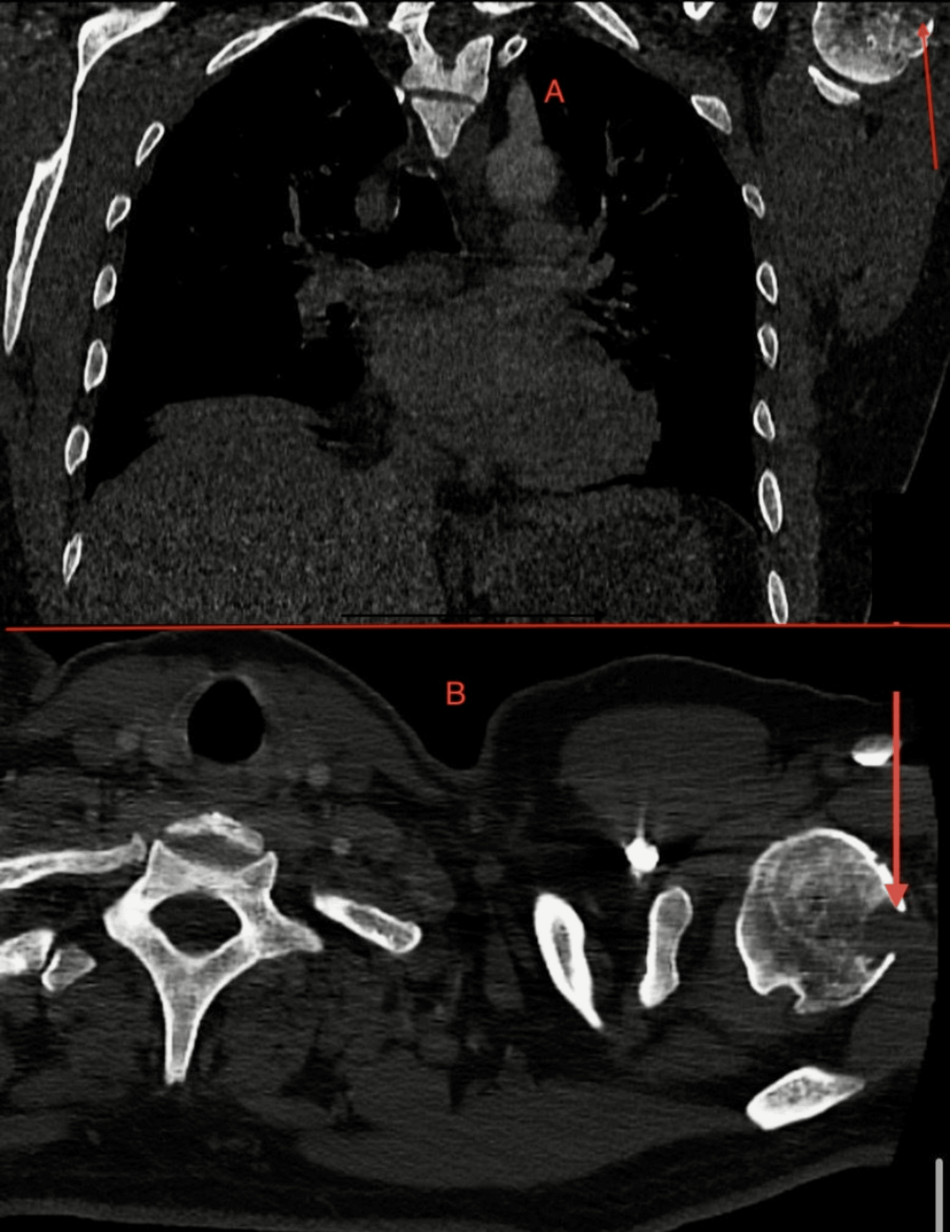
CT of the chest and shoulder demonstrating a 15×11 mm lytic lesion in the left humeral head with cortical rupture A: coronal CT scan showing a bone lesion in the upper thoracic region compatible with granulomatous involvement. B: axial CT scan demonstrating a lytic mass of the right scapula corresponding to osseous sarcoidosis involvement.

Chest and abdominal CT identified a lytic lesion of the left humeral head (15×11 mm) with cortical rupture and sclerotic changes in vertebral bodies (Figure 2).

Laboratory tests showed leukopenia (3600/mm³), lymphopenia, and elevated C-reactive protein (99 mg/dL). Angiotensin-converting enzyme (ACE) was elevated (64.6 U/L), and 24-hour urine calcium was increased. Tests for human leukocyte antigen B27 (HLA-B27), HIV, hepatitis viruses, Brucella, Chlamydia, Coxiella, and tuberculosis were negative.

**Figure 3:**
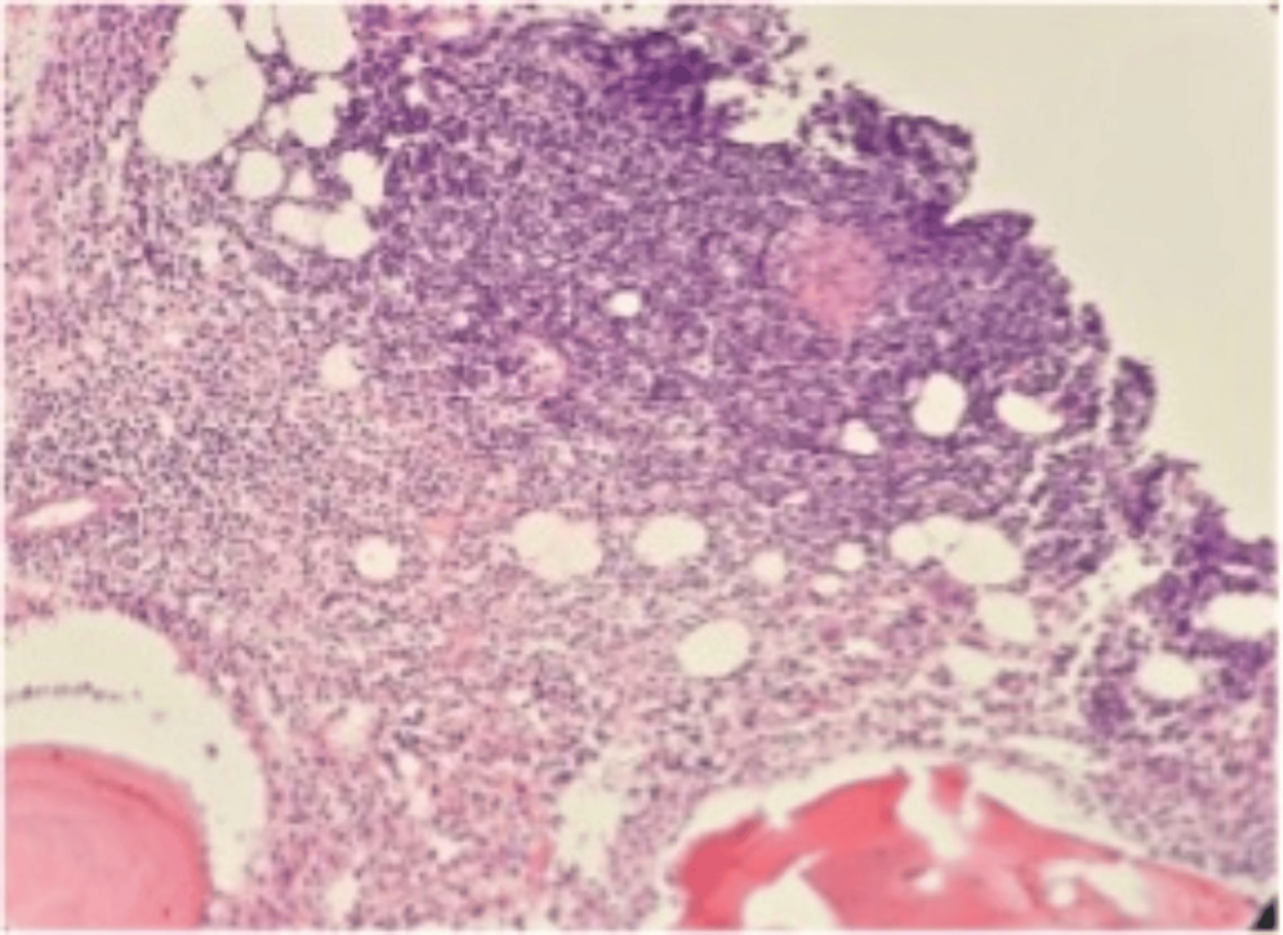
Histological examination of bone biopsy Histological examination of bone biopsy showing non-caseating granulomas composed of epithelioid cells, Langhans-type giant cells, and lymphocytes, without caseous necrosis (H&E staining, ×200).

Bone marrow biopsy and CT-guided biopsy of the iliopsoas lesion revealed non-caseating epithelioid granulomas with Langhans-type giant cells, confirming sarcoidosis (Figure 3).

The patient was started on oral prednisone (1 mg/kg/day) with rapid clinical improvement and significant reduction of pain. A tapering regimen was initiated, with outpatient follow-up scheduled.

## Discussion

Musculoskeletal involvement in sarcoidosis is uncommon but clinically significant. Osseous lesions are reported in 1-15% of cases, often mimicking metastases or infections. Sacroiliac joint disease is particularly rare and has been described in only a limited number of case reports [4-6]. Histological confirmation is essential to distinguish sarcoidosis from tuberculosis or ankylosing spondylitis. Muscle sarcoidosis is usually asymptomatic, though up to 80% of patients show subclinical involvement. Pseudotumoral myositis, as in our case, is the rarest form (~3%) [7-8]. Imaging is useful for lesion detection but nonspecific; biopsy remains the gold standard for diagnosis. Corticosteroids remain the first-line therapy, typically 0.5-1 mg/kg/day with tapering. Methotrexate, azathioprine, or biologic agents targeting tumor necrosis factor alpha (TNF-α)are reserved for refractory cases. Our patient responded well to prednisone, consistent with reported outcomes [9-10].

## Conclusions

Sacroiliac sarcoidosis is a rare manifestation that may mimic inflammatory or malignant bone disease. MRI is crucial for detecting multifocal osseous and muscular lesions, but definitive diagnosis requires histological evidence of non-caseating granulomas. Corticosteroid therapy remains the mainstay of treatment. Early recognition is essential to avoid misdiagnosis and inappropriate therapy.
